# LIGHT/TNFSR14 Can Regulate Hepatic Lipase Expression by Hepatocytes Independent of T Cells and Kupffer Cells

**DOI:** 10.1371/journal.pone.0054719

**Published:** 2013-01-23

**Authors:** Bijoy Chellan, Ekaterina P. Koroleva, Timothy J. Sontag, Alexei V. Tumanov, Yang-Xin Fu, Godfrey S. Getz, Catherine A. Reardon

**Affiliations:** 1 Department of Pathology, University of Chicago, Chicago, Illinois, United States of America; 2 Trudeau Institute, Saranac Lake, New York, United States of America; French National Centre for Scientific Research, France

## Abstract

LIGHT/TNFSF14 is a costimulatory molecule expressed on activated T cells for activation and maintenance of T cell homeostasis. LIGHT over expressed in T cells also down regulates hepatic lipase levels in mice through lymphotoxin beta receptor (LTβR) signaling. It is unclear whether LIGHT regulates hepatic lipase directly by interacting with LTβR expressing cells in the liver or indirectly by activation of T cells, and whether Kupffer cells, a major cell populations in the liver that expresses the LTβR, are required. Here we report that LIGHT expression via an adenoviral vector (Ad-LIGHT) is sufficient to down regulate hepatic lipase expression in mice. Depletion of Kupffer cells using clodronate liposomes had no effect on LIGHT-mediated down regulation of hepatic lipase. LIGHT-mediated regulation of hepatic lipase is also independent of LIGHT expression by T cells or activation of T cells. This is demonstrated by the decreased hepatic lipase expression in the liver of Ad-LIGHT infected recombination activating gene deficient mice that lack mature T cells and by the Ad-LIGHT infection of primary hepatocytes. Hepatic lipase expression was not responsive to LIGHT when mice lacking LTβR globally or only on hepatocytes were infected with Ad-LIGHT. Therefore, our data argues that interaction of LIGHT with LTβR on hepatocytes, but not Kupffer cells, is sufficient to down regulate hepatic lipase expression and that this effect can be independent of LIGHT’s costimulatory function.

## Introduction

LIGHT *(lymphotoxin-like, exhibits inducible expression and competes with HSV glycoprotein D for HVEM, a receptor expressed by T lymphocytes; TNFSF14)* is a costimulatory molecule on T cells that has significant roles in innate and adaptive immune responses [1]. LIGHT is a member of the TNF family of cytokines that has three known receptors, the herpes virus entry mediator (HVEM) expressed predominantly on T cells, lymphotoxin beta receptor (LTβR) found on epithelial and stromal cells and decoy receptor 3, which has been only detected in humans [1], [2]. Hepatocytes and macrophages are among the cells expressing LTβR. LIGHT is also involved in the pathology of various immunological diseases [2], [3]. High levels of LIGHT have been observed in human atherosclerotic plaques and increasing evidence implicates LIGHT in cardiovascular diseases [4]–[6]. We had earlier reported that the T cells in LIGHT transgenic mice (Tg-LIGHT) constitutively expressing LIGHT on T cells (under the control of the lck promoter) were more activated and exhibited increased cytokine secretion [7]. These mice have dramatically reduced hepatic lipase (HL) mRNA levels in the liver and this decrease is dependent upon LTβR signaling [8]. It is unclear if the regulation of HL expression is dependent on the activation state of the T cells or the higher level of LIGHT expression in the Tg-LIGHT mice.

HL is a multifunctional protein whose role in lipoprotein metabolism is well documented [9], [10]. HL catalyzes the hydrolysis of phospholipids and triglycerides in lipoproteins, especially LDL and HDL, and may serve as a bridging molecule that facilitates the uptake of lipoproteins, especially triglyceride-rich lipoproteins, by the LDL receptor related protein. Polymorphisms in the human HL gene contribute to susceptibility to cardiovascular disease, although its effect can be modulated by other lipid abnormalities or polymorphisms in other genes involved in lipoprotein metabolism [11]–[13]. In mouse models, the absence of HL is associated with the appearance of large HDL particles in the plasma and either increased or decreased atherosclerosis, while transgenic expression is associated with increased atherosclerosis [12]. Given that HL activity can influence atherosclerosis susceptibility, understanding what regulates its activity is important.

Macrophages interact with T cells in order to regulate T cell activation in target organs and are themselves activated by cytokines produced by T cells. Macrophages in general have the capacity to synthesize a very large array of molecules which can have a profound influence on other cells. Resident liver macrophages or Kupffer cells are the largest macrophage population in the body [14]. Kupffer cells lie in close proximity to hepatocytes separated by the liver sinusoidal endothelial cells and may be acting as a functional unit capable of complex and tightly regulated interactions. The T cells in Tg-LIGHT mouse are constitutively activated [7] and may potentially be interacting with Kupffer cells. It is unclear also if Kupffer cells have a role in the LIGHT-mediated HL downregulation observed in the Tg-LIGHT mouse [8].

In this study we have examined *in vivo* and *in vitro* whether the LIGHT-mediated inhibition of HL expression is dependent upon LIGHT expression on and activation of T cells and the subsequent secretion of proinflammatory compounds or whether there is direct targeting of liver cells by LIGHT in contrast to the participation of Kupffer cells in LIGHT-mediated regulation of HL. Here we show that adenoviral vector mediated LIGHT expression primarily in the liver inhibits HL expression via interacting with LTβR on hepatocytes and that this effect is independent of T cells. We also show that Kupffer cells, even though they express LTβR, are not required for this regulation. This was demonstrated using animals selectively lacking LTβR in hepatocytes and in mice lacking Kupffer cells due to clodronate liposome treatment. Our results demonstrate that while LIGHT is a T cell costimulatory molecule, HL downregulation by LIGHT is independent of T cells and Kupffer cells indicating a non-costimulatory function for LIGHT.

## Materials and Methods

### Mice

All mice used in this study were backcrossed with C57BL/6 mice for at least 10 generations and maintained under specific pathogen-free conditions. C57BL/6 and LDLR^−/−^ mice were purchased from Jackson Laboratories and bred in our colony. Mice expressing the LIGHT transgene in T cells (Tg-LIGHT) under the control of *lck* promoter and CD2 enhancer were described earlier [7]. LIGHT^−/−^, LTβR^−/−^ [15] and HVEM^−/−^ mice [16] and RAG^−/−^ mice [17] were crossed to LDLR^−/−^ mice and maintained under specific pathogen free conditions. Mice with hepatocyte specific deletion of LTβR gene (H-LTβR^−/−^) were obtained by crossing LTβR floxed mice [18] with albumin-Cre transgenic mice [19] and further backcrossed to LDLR^−/−^ mice. All studies and euthanasia were performed in accordance the Guide for the *Care and Use of Laboratory Animals* and National Institutes of Health guidelines and approved by the University of Chicago Institutional Animal Care and Use Committee.

### Adenovirus Vectors and Mouse Inoculations

The recombinant Ad5 (E1/E3-) adenoviral vector expressing LIGHT (Ad-LIGHT) and null vectors which do not code for any protein (Ad-Null) were generated as described [20]. Recombinant adenoviral construct expressing human apoA-I was generated using Adeno-X expression systems (Clontech). Endotoxin content was assayed using LAL gel clot method **(**Genscript**)** and found to be less than 0.1 ng/µg (1EU/µg). To inoculate mice, 1.25×10^9^ pfu of adenoviral vectors were injected into anesthetized mice through the retro-orbital sinus. Mice were sacrificed on the 7^th^ day post inoculation.

### Kupffer Cell Depletion Using Clodronate Liposomes

Clodronate liposomes (5 mg/ml clodronate) and control liposomes (no clodronate) were purchased from Encapsula Nano Sciences. 200 µl of a 1∶1 PBS diluted liposomes were injected via the tail vein every 5^th^ day for 14 days.

### Real-time PCR

RNA was isolated from liver using TRIzol reagent (Invitrogen). First strand cDNA was generated from 4 µg RNA using Superscript III (Invitrogen) and random primers (Invitrogen). Subsequently, the cDNA was diluted 1∶100 and 2 µl cDNA was subjected to real time PCR using SYBR Green (Qiagen) with specific primers. The primer sequences are: mouse HL forward and reverse primers (5- CTA TGG CTG GAG GAA TCT G -3; 5-TGG CAT CAT CAG GAG AAA G -3), mouse HPRT forward and reverse primers (5- ACC TCT CGA AGT GTT GGA TA-3; 5-CAA CAA CAA ACT TGT CTG GA -3) and primer mix specific for mouse IL-1β (Qiagen). Gene expression was normalized to HPRT.

### Western Blotting and Immunohistochemistry

Total protein was isolated from hepatocytes after isolation of RNA. The protein fraction from the TRIzol extract was acetone precipitated and washed 3 times with 0.3 M guanidine hydrochloride in 1∶1 mixture of 95% ethanol and 2.5% glycerol and finally with ethanol containing 2.5% glycerol (V/V). The washed pellet was then air dried, dissolved in 1% SDS and subjected to SDS PAGE and western blotting using standard techniques. LIGHT was detected using anti-mouse LIGHT (TNFSF14) antibody (R&D systems). Paraffin embedded formalin fixed liver tissue was microtome-sectioned and stained with hematoxylin and eosin (H&E) or for macrophages using anti-mouse F4/80 antibody (1∶500, MCAP497, Serotec). Immunohistochemistry tissue sections were incubated with the primary antibody for 1 hour at room temperature followed by biotinylated anti-rat IgG (10 µg/ml, BA-4001, Vector laboratories, CA, USA) for 30 minutes at room temperature. The antigen-antibody binding was detected by the Elite kit (PK-6100, Vector Laboratories) and the DAB (DAKO, K3468) system. Alanine aminotransferase (ALT) was measured according to manufacturer’s instructions (Cayman Chemicals).

### Hepatocyte Isolation, Purification and Infection with Adenovirus

Mouse hepatocytes were isolated by a method modified from that described by Sacci JB [21]. Mouse livers were perfused through the left ventricle of the heart and the portal vein was cut to allow the exit of the perfusate. Perfusion with HBSS (without Ca^++^ and Mg^++^) containing EDTA and HEPES (0.5 and 10 mM respectively) for 10 minutes was followed with HBSS containing 276 U/ml collagenase (Type IV, Sigma), 3.73 mM CaCl_2_ and 10 mM HEPES for 10 minutes. The isolated hepatocytes were then mixed with isosmotic Percoll at 1∶1 (total volume 20 ml) and centrifuged at 50G for 20 minutes at 4°C. Viable cells at the bottom of the tube were washed with HBSS, centrifuged at 50G and resuspended in complete Williams E medium (10% FBS, 1% PSG). Cell viability was more than 95%, with yields ranging from 15–20×10^6^ hepatocytes from a single mouse liver. Cells were plated at a density of 1×10^6^ cells/well on rat tail collagen (Type I, BD Biosciences) coated 6 well plates. After 4 hours, the plates were washed with PBS, the media removed and viral vectors were added to the wells at a multiplicity of infection (MOI) of 5 in 500 µl serum-free Williams E media and incubated at 37°C for 3 hours. The cells were washed with PBS and incubated for up to 36 hours at 37°C with complete Williams E media. To isolate nonparenchymal cells, following collagenase digestion of the liver, the cells were centrifuged at 50G for 5 minutes at 4°C. The pellet was discarded and the supernatant, which contains the nonparenchymal cells, was washed thrice in PBS before resuspending in complete Williams E medium. In coincubation experiments, nonparenchymal cells were used at 5×10^6^ cells per well.

### FL83B cell-Ad-virus Transduction and Hepatocyte Coculture

FL83B cells (mouse liver cell line) purchased from ATCC **(**CRL-2390) was plated in collagen coated 6 well plates at a density of 0.3×10^6^ cells/well in complete Williams E media. After 12 hours, the media was removed and viral vectors were added to the wells at an MOI of 5 in 500 µl serum-free Williams E-media and incubated for 3 hours. The cells were washed with PBS and incubated for an additional 3 hours at 37°C with complete Williams-E media. Freshly isolated hepatocytes were then added to the wells containing the FL83B cells at a density of 0.5×10^6^ cells/well in 1 ml fresh complete Williams E media. The hepatocytes adhered to the wells, forming a confluent layer intermingled with the FL83B cells after about 12 hours. The coculture was incubated for 36 hours at 37°C.

### Statistical Analysis

Results are presented as mean ± standard deviation**.** Statistical differences were analyzed using two tailed Student’s *t*-test. *P<*0.05 was considered significant.

## Results

### Depletion of Kupffer Cells has No Effect on HL Down Regulation by LIGHT

**Figure 1:**
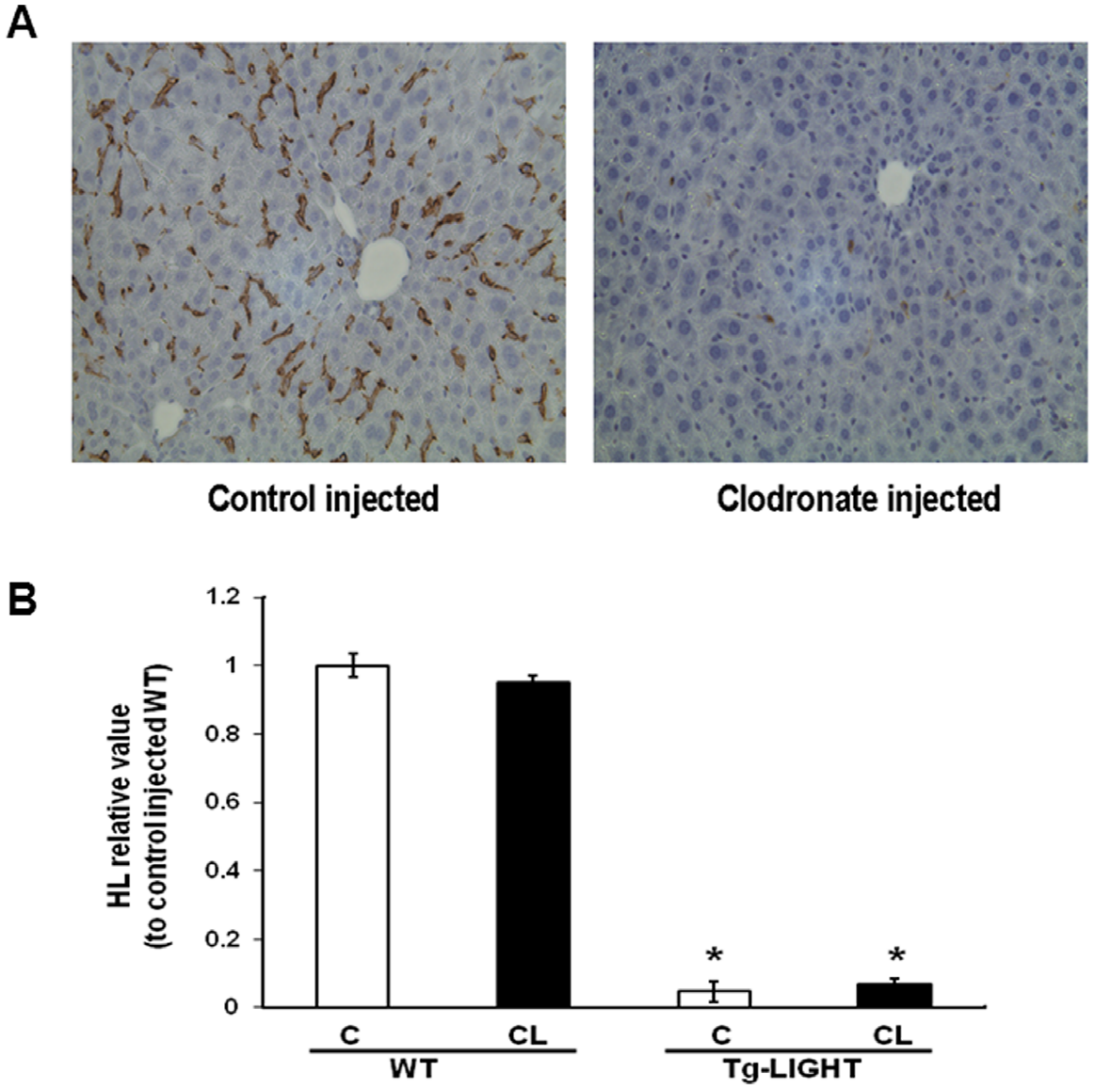
LIGHT-mediated HL regulation in mice is independent of the presence of Kupffer cells. To deplete Kupffer cells, liposomes containing clodronate were injected through the tail vein into wild type C57BL/6 mice or Tg-LIGHT mice every 5^th^ day for 14 days. Control liposomes did not contain clodronate. (A) F4/80 staining for liver Kupffer cells in control liposome and clodronate liposome injected Tg-LIGHT mice. (B) Real-time PCR data for HL mRNA expression in the liver of control (C) and clodronate (CL) liposome injected wild type (WT) and Tg-LIGHT mice. (n = 3; *p<0.01 WT vs. Tg-LIGHT).

We have previously shown that the transgenic over expression of LIGHT in T cells down regulates HL expression in the liver [8]. In these mice, there was a dramatic decrease in HL mRNA levels in the liver, as well as decreased plasma HL activity and protein. There was no difference in the levels of apoE or apoA-I mRNA (data not shown) indicating that the expression of LIGHT was not influencing all aspect of liver lipoprotein metabolism. T cells are known to interact with Kupffer cells [22] and Kupffer cells interact with hepatocytes to regulate some of their functions, especially in inflammatory conditions [23], [24]. To exclude a role for Kupffer cells *in vivo* in the LIGHT mediated regulation of HL, we depleted Kupffer cells in Tg-LIGHT mice using clodronate liposomes. Clodronate is taken up by phagocytic cells leading to apoptosis. This ‘suicide’ approach is highly effective in depleting Kupffer cells in liver tissue [25], [26], with Kupffer cells being depleted as early as 12 hours after injection and not reappearing until the 4^th^ day [27]. Clodronate released from dead macrophages or by leakage from liposomes will not induce systemic toxicity since it is only taken up by cells by phagocytosis and will not cross cell membranes. It has an extremely short half-life in circulation and body fluids [27]. Following the injection of clodronate liposomes every 5^th^ day for 14 days there was almost complete depletion of Kupffer cells in the liver of Tg-LIGHT mice (Fig. 1A). However, HL mRNA levels were down regulated in Tg-LIGHT mice regardless of whether they received control or clodronate liposomes (Fig. 1B). Also, even in wild type mice Kupffer cell depletion had no effect on liver HL mRNA levels. The data suggests that Kupffer cells do not have a role in HL down regulation observed in Tg-LIGHT mice.

These studies on the role of LIGHT regulating HL expression in the liver were initiated as part of our studies examining the role of LIGHT and its receptors on atherosclerosis. In these studies we are using the atherosusceptible LDL receptor deficient (LDLR^−/−^) mice. We have shown that the transgenic expression of LIGHT in the absence of the LDLR also profoundly decreases HL expression. Thus to be consistent with the atherosclerosis studies, in most of the rest of the studies reported here we used mice in the LDLR^−/−^ background.

### HL Expression in Mice Expressing LIGHT via an Adenoviral Vector: Lack of a Role for Kupffer cells, T cells and IL-1β

**Figure 2:**
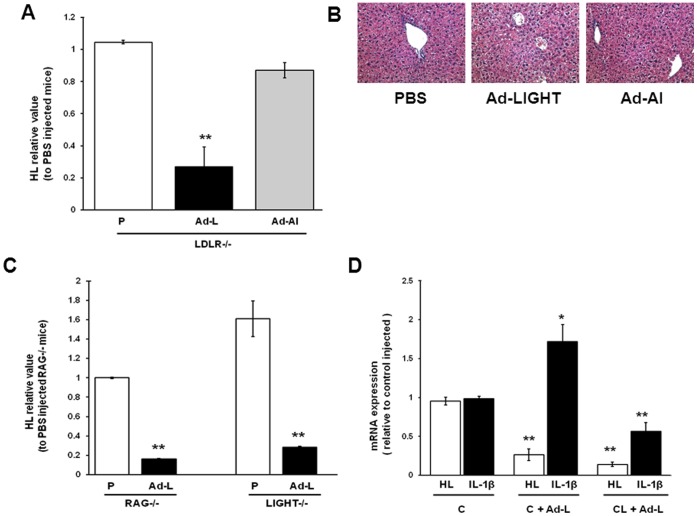
Decreased HL expression in Ad-LIGHT infected mice is independent of T cells and IL-1β. Mice were injected with PBS or adenoviral vectors (1.25×10^9^ pfu/mouse) and sacrificed on the 7^th^ day. (A) Liver HL mRNA expression in LIGHT adenovirus (Ad-L), human apoA-I adenovirus (Ad-AI) or PBS (P) injected LDLR^−/−^ mice was analyzed by real time PCR. (B) Morphology of liver from PBS and adenoviral infected mice (H & E staining, 20x objective). (C) Liver HL mRNA expression in Ad-L and P injected RAG^−/−^LDLR^−/−^ and LIGHT^−/−^LDLR^−/−^ mice. (D) Liver HL and IL-1β mRNA expression in Ad-L injected LDLR^−/−^ mice treated with control (C) or clodronate (CL) liposomes. The virus was injected 2 days after clodronate injection. (n = 3; *p<0.05, **p<0.01; for panels A and C: vs. PBS treated mice; for Panel D: vs. control liposome treated mice.).

In the transgenic mice, LIGHT on the T cells may be interacting directly with its receptors on hepatocytes to mediate the decrease in HL expression. Alternatively, other cell surface proteins on the activated T cells or proteins secreted by the activated T cells may be mediating the effect on HL expression. To determine if LIGHT expression specifically by T cells was obligatory for LIGHT- mediated down regulation of HL, LDLR^−/−^ mice were injected with an adenoviral vector expressing mouse LIGHT (Ad-LIGHT) or a control virus expressing human apoA-I (Ad-AI). Adenoviruses injected intravenously are primarily taken up by the liver. As shown in Fig. 2A, adenoviral mediated expression of LIGHT was sufficient to induce a down regulation of HL. The adenoviral mediated expression of apoA-I had no effect on HL expression indicating that the effect of Ad-LIGHT on HL expression is not likely due to the effect of adenoviral infection in general. The LIGHT effect was not due to increased liver toxicity as determined by similar by alanine aminotransferase activity (10 U/L) and morphology (Fig. 2B).

Recombinant adenoviral expression is known to activate T cells without infecting them directly [28] and endogenous LIGHT expression is increased in activated T cells [1], [2]. To examine if Ad-LIGHT was causing HL down regulation through increased expression of endogenous LIGHT on T cells, we injected Ad-LIGHT to LIGHT deficient mice (LIGHT^−/−^) mice. Ad-LIGHT decreased HL expression in LIGHT^−/−^ mice (Fig. 2C) indicating that the Ad-LIGHT mediated down regulation of HL expression is independent of T cell expression of endogenous LIGHT.

To further rule out the participation of other surface molecules or cytokines secreted by T cells, we injected Ad-LIGHT into recombination activating gene (RAG) deficient mice which lack both mature T and B cells [29]. HL was down regulated to levels observed in the Tg-LIGHT mice in these immune incompetent mice (Fig. 2C). All together, the data indicate that the down regulation of HL by LIGHT does not specifically require that LIGHT be expressed by the T cells nor does it require the cytokines or other products produced by activated. Thus LIGHT may be able to directly signal to hepatocytes, HL is known to be down regulated by a variety of cytokines including IL-1β [30]. Kupffer cells are a major source of IL-1β in the liver [31] and IL-1β expression is increased following adenoviral infection [32] and other inflammatory situations (e.g. ischemia-reperfusion, activation of TLR9) [33], [34]. To determine if IL-1β is involved in Ad-LIGHT induced HL down regulation we injected Ad-LIGHT into wild type mice with and without prior Kupffer cell depletion. As expected hepatic IL-1β mRNA levels were elevated in the Ad-LIGHT injected mice and this level decreased significantly after Kupffer cell depletion (Fig. 2D). However, the Ad-LIGHT mediated decrease in HL mRNA levels was not affected by Kupffer cell depletion. This result suggests that Ad-LIGHT down regulation of HL expression in mice is independent of IL-1β expressed by Kupffer cells. Another member of the TNF superfamily that LIGHT belongs to, namely TNFα, has been shown previously not to regulate HL expression [30] and we did not observe an effect of Ad-LIGHT infection on hepaticTNFα mRNA levels (data not shown).

### HL Expression in Mice Expressing LIGHT via an Adenoviral Vector: Role of Receptors

**Figure 3:**
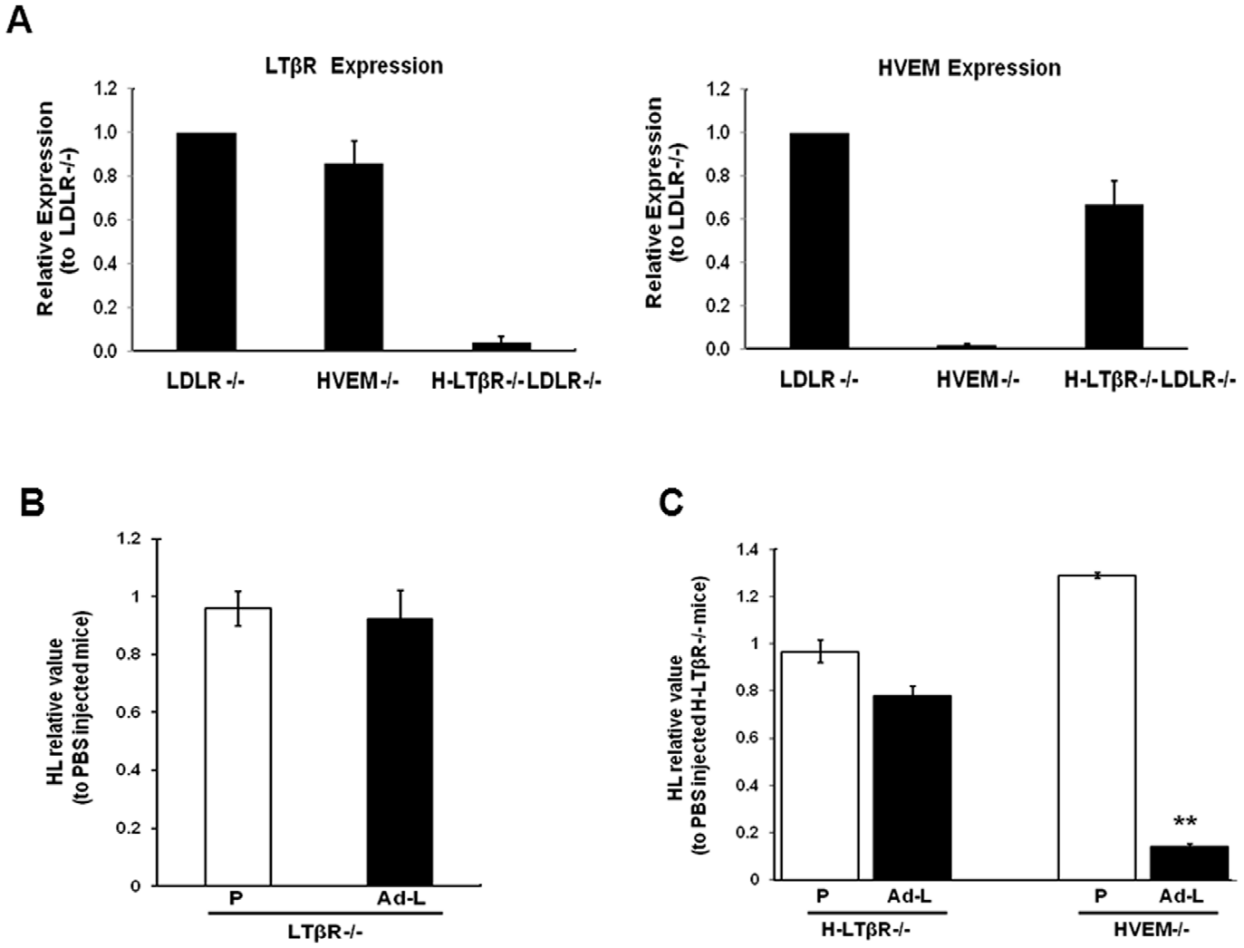
LTβR expression on hepatocytes is sufficient for HL regulation by Ad-LIGHT. (A) Liver LTβR and HVEM expression in LDLR^−/−^, HVEM^−/−^LDLR^−/−^ and hepatocyte-specific knockout of LTβR (H-LTβR^−/−^) LDLR^−/−^ mice. (B and C) Mice were injected with PBS or adenovirus and sacrificed on the 7^th^ day. Liver HL mRNA expression in Ad-LIGHT (Ad-L) and PBS (P) injected LTβR^−/−^LDLR^−/−^ mice (B) and (H-LTβR^−/−^LDLR^−/−^ and HVEM^−/−^LDLR^−/−^ mice C). (n = 3; **p<0.01 vs. PBS injected mice).

LIGHT interacts with two cell surface receptors in mice, namely LTβR and HVEM. Hepatocytes express LTβR [15], whereas Kupffer cells express LTβR and may express HVEM as they are derived from the myeloid lineage [4], [14], [35], [36]. To explore the role of LIGHT receptors, we injected Ad-LIGHT into HVEM^−/−^LDLR^−/−^ mice, LTβR^−/−^LDLR^−/−^ mice, and LDLR^−/−^ mice with LTβR inactivated specifically in hepatocytes (H-LTβR^−/−^ LDLR^−/−^). Previous studies have demonstrated the absence of LTβR in the liver of LTβR^−/−^ mice [37]. As seen in Fig. 3A, HVEM expression is absent in the liver of HVEM^−/−^ LDLR^−/−^ mice and LTβR expression is absent in the liver of H-LTβR^−/−^LDLR^−/−^ mice. While Ad-LIGHT decreased HL expression in LDLR^−/−^ mice (Fig. 2A), there was no statistically significant decrease in HL expression in Ad-LIGHT injected LTβR^−/−^LDLR^−/−^ mice (Fig. 3B) or H-LTβR^−/−^LDLR^−/−^ mice (Fig. 3C). On the other hand, Ad-LIGHT induced significant HL down regulation in HVEM^−/−^LDLR^−/−^ mice (Fig. 3C). These results indicate that LIGHT interacts with LTβR on hepatocytes to inhibit HL expression.

### LIGHT can Directly Interact with LTβR on Hepatocytes to Decrease HL Expression

**Figure 4:**
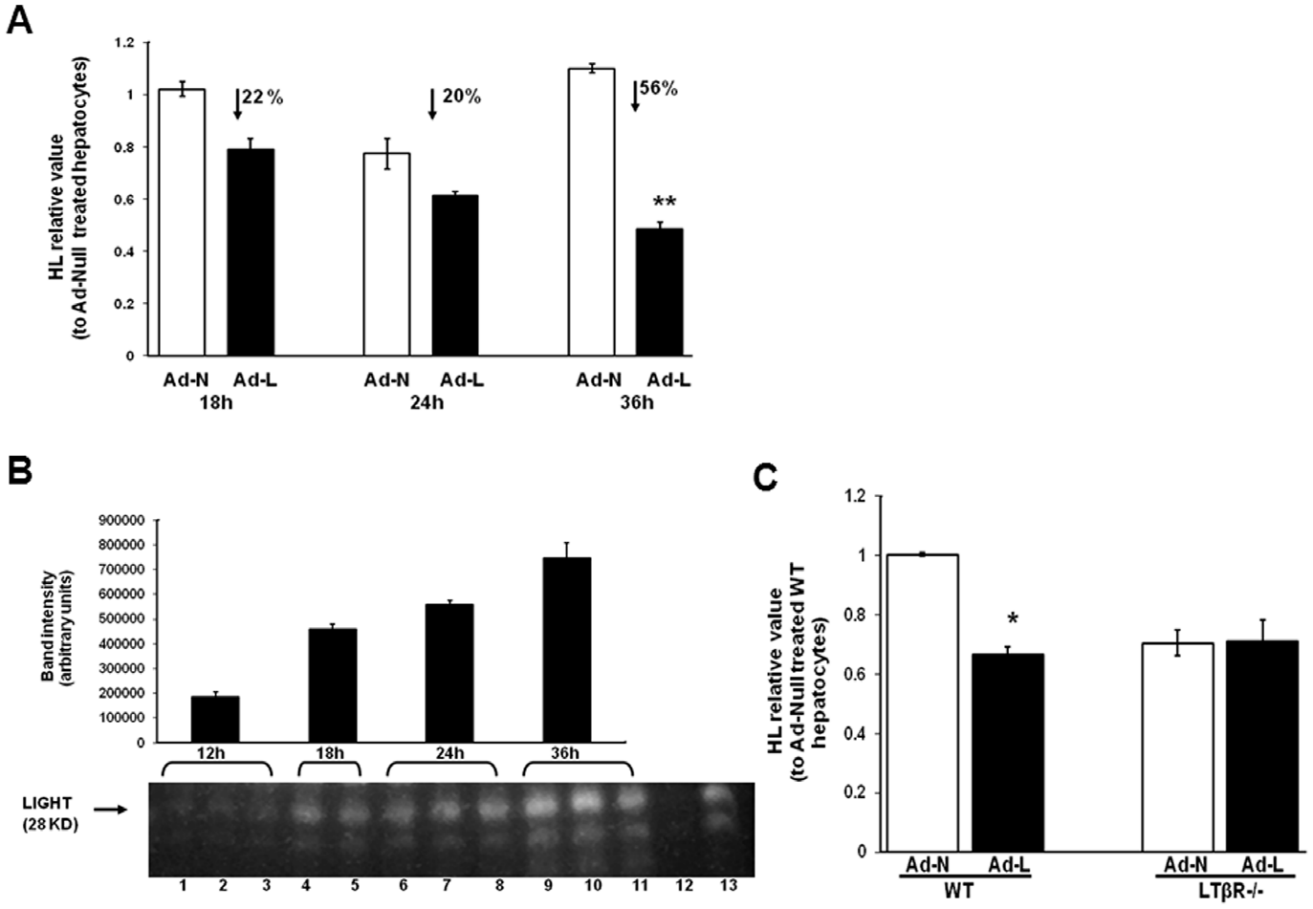
LIGHT and HL expression in Ad-LIGHT transduced primary hepatocytes in vitro. (A) HL mRNA expression in Ad-LIGHT (Ad-L) and Null adenovirus (Ad-N) infected primary hepatocytes from wild type mice 18, 24 and 36 hours post-infection. The numbers indicate the % decrease in expression in the Ad-LIGHT infected cells relative to the Ad-N infected cells. (B) Total protein from 1×10^6^ Ad-LIGHT infected hepatocytes (in triplicate) at various times post-infection were immunoblotted with anti-mouse LIGHT antibody. Lane 12 is from non-infected hepatocytes and lane 13 is from Tg-LIGHT mouse spleen. (C) Liver HL mRNA expression in Ad-N and Ad-L infected hepatocytes from LDLR^−/−^ and LTβR^−/−^LDLR^−/−^ mice. (n = 3; *p<0.05, **p<0.01 vs. Ad-N).

To further establish that Ad-LIGHT mediated HL down regulation is due to direct interaction of LIGHT with LTβR on hepatocytes, isolated primary hepatocytes from LDLR^−/−^ mice were infected with Ad-LIGHT or a null vector (Ad-Null). HL mRNA expression by hepatocytes was found to be down regulated significantly over a period of 36 hours post-infection with Ad-LIGHT (Fig. 4A). During this time LIGHT protein expression by Ad-LIGHT infected hepatocytes progressively increased (Fig. 4B). When LIGHT was expressed in LTβR-deficient hepatocytes, there was no HL down regulation (Fig. 4C), consistent with our previous observations about the involvement of LTβR in LIGHT-mediated regulation of HL.

**Figure 5:**
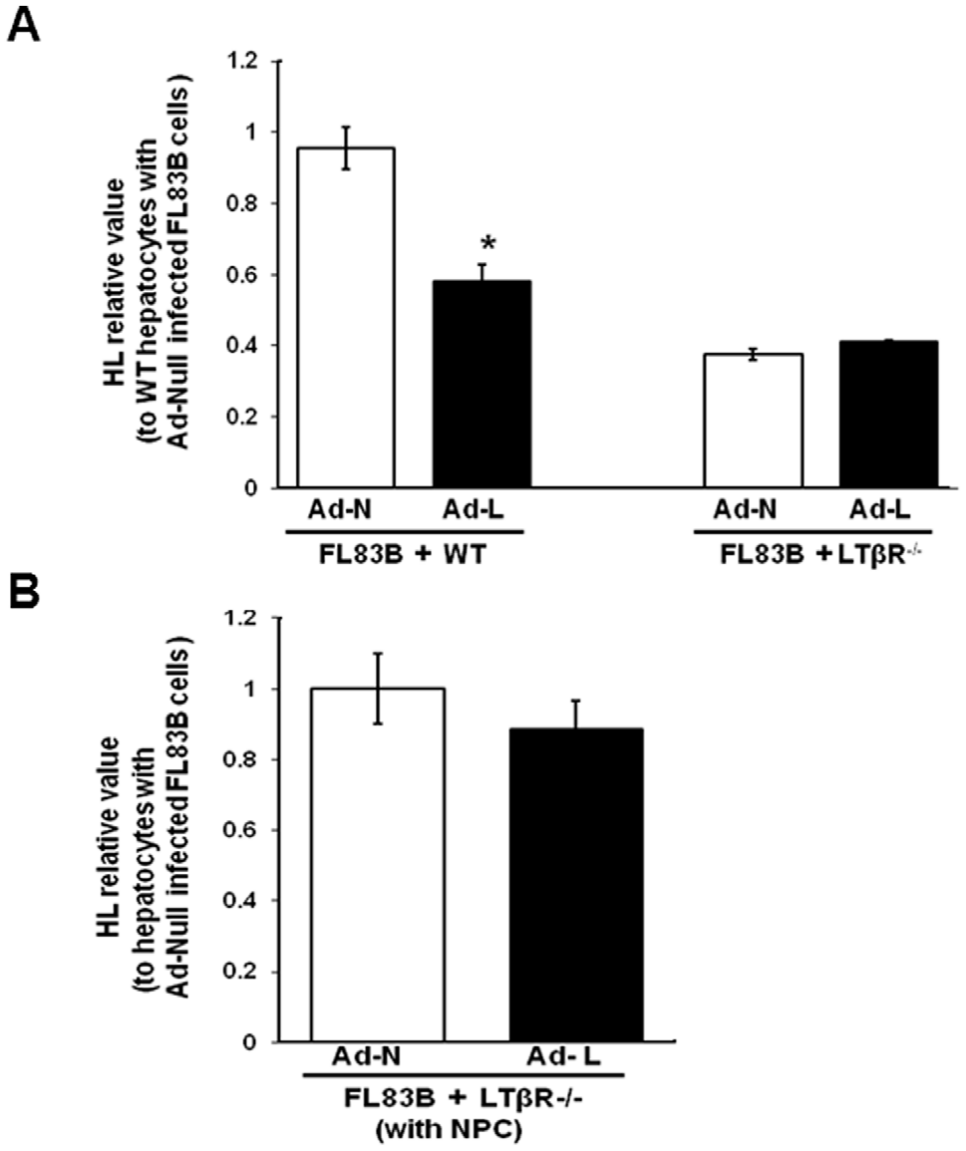
Evidence for trans-regulation by LIGHT and lack of a role of liver non-parenchymal cells. (A) HL mRNA expression in hepatocytes from LDLR^−/−^ (WT) and LTβR^−/−^LDLR^−/−^ mice cocultured with Ad-LIGHT (Ad-L) or Ad-Null (Ad-N) infected FL83B cells. (B) HL mRNA expression in hepatocytes from LTβR^−/−^LDLR^−/−^ mice cocultured with Ad-N or Ad-L infected FL83B cells and Tg-LIGHT mouse liver non-parenchymal cells (NPC). (n = 3; *<0.05 vs. Ad-N infected FL83B coculture).

LIGHT is a cell surface molecule. To establish if HL downregulation by LIGHT in our hepatocyte culture system requires LIGHT to be expressed in the HL expressing cell, we infected FL83B cells, which do not produce HL (data not shown), with Ad-LIGHT and coincubated the infected cells with uninfected primary hepatocytes from LDLR^−/−^ or LTβR^−/−^LDLR^−/−^ mice. Coincubation of LIGHT expressing FL83B cells with primary hepatocytes from mice expressing LTβR significantly reduced HL expression but not when coincubated with primary hepatocytes from LTβR^−/−^ mice (Fig. 5A). To further rule out the role of non-parenchymal cells expressing LTβR, non-parenchymal cells from Tg-LIGHT mice were coincubated with Ad-LIGHT infected FL83B cells and primary hepatocytes from LTβR^−/−^ mice. The inclusion of non-parenchymal cells from the Tg-LIGHT mice was without an effect on the lack of down regulation of HL upon coincubation with hepatocytes lacking LTβR in the presence of LIGHT expressing FL83B cells (Fig. 5B). These results are consistent with the concept that LIGHT-mediated HL down regulation operates via direct interaction of LIGHT with LTβR on hepatocytes.

## Discussion

In this work we show that LIGHT over expression *in vivo* down regulates the expression of HL in hepatocytes in an LTβR-dependent manner that is independent of Kupffer cells, T cells, and endogenous T cell expression of LIGHT. HL is synthesized primarily in the liver by hepatocytes and is regulated as a function of cholesterol homeostasis and several physiological and pharmacological factors [9] and by inflammatory factors such as IL-1β [30], autoimmunity [38] and the acute phase response [39], [40]. T cell activation is a hallmark of inflammatory responses and LIGHT is expressed on T cells during activation. We previously reported that HL expression is decreased in a mouse model constitutively expressing LIGHT on T cells (Tg-LIGHT mice) [8]. Kupffer cells and T cells are two primary components of the inflammatory phenotype in the liver. In our present experiments we aimed to establish whether Kupffer cells or T cells had an independent role in the HL down regulation observed in Tg-LIGHT mice. We established that LIGHT directly reduced hepatocyte HL expression without any obligatory contribution of Kupffer cells and T cells. Our data suggests that the interaction of LIGHT with LTβR on hepatocytes is sufficient to inhibit HL expression in the absence of other cell interactions (i.e. T cell-Kupffer cell, T cell-hepatocyte, or Kupffer cell-hepatocyte) or T cell products.

Kupffer cells, the liver resident macrophages, play an important role in the acute and chronic responses of the liver to toxic compounds. Activation of Kupffer cells results in the release of an array of inflammatory and growth control mediators that have profound impact on hepatocytes [21] and contributes to hepatic inflammation. Since Kupffer cells express LTβR, it is possible that LIGHT-mediated activation of Kupffer cells is responsible for the down regulation of HL expression by hepatocytes. However, there was no HL downregulation in Ad-LIGHT injected mice that lack LTβR expression in hepatocytes, but express LTβR in Kupffer cells. This indicates that LTβR on hepatocytes is sufficient for LIGHT- mediated HL down regulation. The lack of a role of Kupffer cells was also demonstrated by the absence of an effect on HL expression upon depletion of Kupffer cells in Tg-LIGHT mice using clodronate, by the down regulation of HL in Ad-LIGHT infected primary hepatocytes in culture and in the coincubation studies using LIGHT expressing FL83B hepatoma cells. This latter study also indicates that that LIGHT-mediated HL down regulation operates via direct interaction of LIGHT with LTβR on hepatocytes.

Several studies have demonstrated that hepatocytes are efficient antigen presenting cells *in vitro* [41], [42] as well as *in *vivo [43], [44], with significant relevance to hepatotropic viral infections such as hepatitis C. There is a strong likelihood for T cell activation and possible T cell-hepatocyte interactions in our adenoviral vector injection experiments. LIGHT is normally expressed on activated T cells and immature dendritic cells [1], [2]. However the experiments in which HL mRNA was significantly decreased upon injection of Ad-LIGHT into RAG^−/−^ mice lacking T cells and the coculture of LIGHT expressing FL83 cells with primary hepatocytes exclude the requirement of other aspects of T cell activation (e.g. secretion of cytokines) for the LIGHT-mediated regulation of HL. The role of various T cell subsets in LIGHT-mediated down regulation of HL remains unknown. Natural killer T (NKT) cells are a major T cell population within the liver and are CD1d restricted. Therefore, mice deficient in CD1d are NKT cell deficient. We found that the expression of the LIGHT transgene in T cells in CD1d^−/−^ mice had a similar effect on HL expression levels as in Tg-LIGHT mice (data not shown).

LIGHT is a cell surface protein that functions as a costimulatory molecule. While in our transgenic mouse model and in the adenoviral infected mice there is little soluble LIGHT as its cleavage site has been mutated in the transgene [7], in humans, for example, LIGHT can be cleaved from the cell membranes by metalloproteases. Soluble LIGHT is found in increased levels in the plasma of atherosclerotic patients [6] and monocyte/macrophages, dendritic cells or T cells may be the source of the soluble LIGHT. LIGHT has also found to be associated with platelets and released as a soluble ligand upon platelet activation [45]. The possible existence and action of soluble LIGHT is consistent with our results indicating that LIGHT can function independently of T cells and T cell costimulation to directly regulate HL expression in the liver.

Our results indicate that LIGHT interaction with the LTβR on hepatocytes is responsible for the down regulation of HL expression. Lymphotoxin β is also able to signal via the LTβR and it is possible that this ligand may also regulate HL expression. Lymphotoxin β is a heterotrimer of LTβ and LTα and thus to overexpress this ligand would require increased expression of both subunits. This will complicate the analysis since the homotrimer of LTα will also be generated. This ligand does not interact with the LTβR, but rather interacts with a different set of receptors of the TNFα superfamily.

HL levels in the liver of LIGHT^−/−^ or LTβR^−/−^ mice under basal conditions are similar to those in wild type animals. This is perhaps not surprising given that LIGHT expression in the absence of inflammation is low. HL expression is decreased in the acute phase response [39], [40]. However, upon induction of the acute phase response in LIGHT^−/−^ mice with lipopolysaccharide, we observed a decrease in HL expression despite the absence of LIGHT (data not shown). Again this is perhaps not surprising since a number of cytokines are increased in the acute phase response including IL-6 and IL-1β that regulate hepatic lipase expression. Thus we suggest that LIGHT is one of the regulators of HL expression, but clearly not the only one.

The mechanism by which LIGHT regulates HL expression is not clear. We have shown that the mRNA levels for two transcription factors that regulate HL expression, HNF1 and HNF4 [46], are not altered in the liver of Tg-LIGHT mice (data not shown). This does not however preclude differences in the activation of the transcription factors. The LTβR has been shown to activate both the classical and noncanonical NFκB pathways and weakly the JNK pathways [47], but whether HL expression is influenced by these signaling pathways is not currently known. In endothelial cells, cytokine mediated upregulation of another member of the lipase family, endothelial lipase, is apparently via NFκB [48]. Further studies are needed in order to determine the involvement of any of these pathways, either directly or indirectly, in the LIGHT/LTβR mediated suppression of HL expression by hepatocytes.

HL has both a ligand function that participates in the hepatic recognition of triglyceride-rich lipoproteins and a catalytic function that participates in the remodeling of LDL and HDL to smaller and denser particles. Depending upon the underlying lipoprotein phenotype, alterations in HL expression may increase or decrease coronary artery vascular disease [49]. Decreased HL may contribute to increased cardiovascular disease in patients with isolated hypercholesterolemia, but with normal triglyceride levels and buoyant LDL particles. The mechanism by which LIGHT impacts cardiovascular disease is not clear, but may in part be related to its modulation of HL expression. Thus it is important to understand the relationship between LIGHT and HL expression.
